# Factors Associated with COVID-19 Vaccine Hesitancy among People with Epilepsy in Lithuania

**DOI:** 10.3390/ijerph18084374

**Published:** 2021-04-20

**Authors:** Kristijonas Puteikis, Rūta Mameniškienė

**Affiliations:** 1 Faculty of Medicine, Vilnius University, 03101 Vilnius, Lithuania; Kristijonas.puteikis@mf.stud.vu.lt; 2 Center for Neurology, Vilnius University, 08661 Vilnius, Lithuania

**Keywords:** COVID-19, epilepsy, influenza, vaccine hesitancy

## Abstract

The purpose of our study was to determine the willingness to be vaccinated against COVID-19 and factors associated with vaccine hesitancy among people with epilepsy (PWE). In December 2020, we performed an online cross-sectional survey of PWE and their caregivers in Lithuania before the rollout of COVID-19 vaccines to the public. The study sample consisted of 111 respondents (44 (39.6%) male, median age 25 years (range 1 to 70)). From 58 PWE who personally responded to the survey, 27 (46.6%) would be willing to be vaccinated against COVID-19. Among the 53 caregivers, 18 (34.0%) would accept the person they care for to be vaccinated. Willingness to be vaccinated was associated with receiving an influenza shot in 2020 (odds ratio (OR) = 9.17, 95% confidence interval (CI = 1.15–73.47), the beliefs that vaccines are generally safe (OR = 7.90, 95% CI = 2.43–25.74) and that they are the only convenient way to gain immunity (OR = 3.91, 95% CI = 1.02–15.05). Respondents were hesitant to accept the COVID-19 vaccine if they thought it could cause the infection (OR = 0.14, 95% CI = 0.04–0.49). COVID-19 vaccine hesitancy is frequent among PWE and their caregivers. It is probably related to erroneous beliefs about their safety and mechanism of action.

## 1. Introduction

The global threat of COVID-19 has greatly influenced the daily lives and health status of people with epilepsy (PWE). A deterioration of their physical and psychological condition was mostly associated with a lack of timely medical services, disrupted use or supply of antiseizure drugs (ASDs) and the consequences of strict lockdown measures, which induced mental health problems and were associated with seizure exacerbation [1,2,3,4,5]. While there is currently only limited evidence that COVID-19 is more severe or lethal in those with comorbid epilepsy, the spread of COVID-19 still exposes patients to a substantial risk of severe respiratory complications and often results in lockdowns, which disturb access to healthcare [6,7,8]. There were two periods of lockdown in Lithuania in 2020 (March–June and November–December) [9]. However, the death rate from COVID-19 increased significantly only towards the end of the year (reaching up to 60 deaths per day for a population of 2.8 million). It is therefore important to know how PWE perceive vaccination and whether they would agree to be vaccinated against COVID-19 to protect themselves and their communities. We report a survey of PWE and their caregivers in which we aimed to determine their willingness to be vaccinated against COVID-19 and define variables (e.g., vaccination history or reported beliefs) that are associated with vaccine hesitancy.

## 2. Materials and Methods

We conducted an online cross-sectional anonymous survey among PWE and their caregivers in Lithuania from 7 December to 31 December 2020. The questionnaire (Supplementary File S1) was created in the survey administration platform Google Forms (Google Inc., Mountain View, CA, USA) and distributed through two social media channels, both of which unite PWE and their caregivers in Lithuania. The first was an official page of a non-governmental patient organization and the second was a closed social networking group dedicated only for epilepsy-related communication among group members.

The survey was divided into three parts: (1) general and clinical characteristics of PWE, (2) COVID-19 infections among PWE and (3) the respondents’ outlook on vaccines. While most adult (≥18 years old) PWE indicated their personal outlook on vaccination, caregivers were asked to provide information about the person they care for as their legal guardians (e.g., for minors or developmentally disabled adults). That is, caregivers specified clinical information about the patient, but reported their own view on vaccination and any decisions they would make as the patient’s representative.

No personal information (e.g., name, email, address or phone number) was collected during the study. The respondents were informed that participation in the survey was greatly appreciated, that only anonymous data were collected, and that the completion of the questionnaire would take up to ten minutes. To avoid missing data, it was required to answer each question before submission. All completed surveys were used for analysis (the only exclusion criteria was being <18 years old if self-identified as a PWE). According to local regulations, such an anonymous online survey did not require approval from the Regional Bioethics Committee.

All statistical analysis was conducted in Microsoft Excel 16.0 (Microsoft) and SPSS Statistics 23.0 (IBM). Categorical data were compared by using Chi-square and Fisher’s exact tests. Ordinal and non-normally distributed continuous variables were analyzed by using Mann–Whitney U and Kruskal–Wallis tests (comparison of independent samples) and Spearman’s rho (correlation). Wilcoxon’s rank test was used when comparing the rate of seizures in the summer of 2020 (relaxed national COVID-19 restrictions and low infection numbers) and seizure frequency in December 2020 (imposed national COVID-19 restrictions and high infection numbers). Only the rates of those persons who experience at least several seizures per month were comparable in this part of the analysis. A binary logistic regression model was created to determine relevant variables associated with the willingness to be vaccinated against COVID-19.

## 3. Results

### 3.1. Survey Participants

General and clinical characteristics of PWE (indicated by them personally or their caregivers) are presented in Table 1. Caregivers provided information for persons who were younger (*p* < 0.0001)—just six patients (11.3%) represented in the survey by their caregivers were adults (≥18 years). Among all patients, 38 (34.2%) were on ASD monotherapy and 68 (61.3%) were on polytherapy (the number of ASDs reported did not differ between the patient and the caregiver groups, *p* = 0.147). After a consultation by a specialist in neurology, the ASD regimen was changed for 47 (42.3%) patients during the COVID-19 pandemic. In eight (7.2%) cases, the use of ASDs was modified by the patients themselves or their caregivers with no supervision of the treating neurologist.

Among persons who experience at least several seizures per month (*n* = 57, 51.4%), seizure frequency was not significantly different in the summer of 2020 as opposed to December 2020 (Wilcoxon signed ranks Z = −0.302, *p* = 0.763 for both the patient and the caregiver groups). Overall, eight (14.0%) of these individuals reported higher seizure frequency and eight (14.0%) indicated a lower rate of their seizures during summer.

Eight (7.2%) persons with epilepsy in our study were diagnosed with COVID-19 and experienced mainly fever (*n* = 6), fatigue (*n* = 5) or headache (*n* = 5). One patient indicated an increase in seizure frequency (by less than two times) when being ill with COVID-19, while others experienced seizures at a usual rate. None were hospitalized or changed their ASD regimen when being ill; one respondent was consulted by an epileptologist and went to the emergency department.

### 3.2. Outlook on Vaccines

The score (on a scale from 1 to 10) of how much participants are waiting for a COVID-19 vaccine (Table 2) was not associated with the age or sex of the patient (Spearman’s rho = 0.026, *p* = 0.788, Mann–Whitney U = 1355.5, *p* = 0.461, respectively), seizure type (Kruskal–Wallis Chi square = 3.407, *p* = 0.492) or being infected with COVID-19 (Mann–Whitney U = 402.5, *p* = 0.911). It was related to the number of ASDs the patient uses (Spearman’s rho = 0.193, *p* = 0.043) and significantly higher in the group that would agree with vaccination against COVID-19 (Mann–Whitney U = 223.0, *p* < 0.0001). The willingness to be vaccinated for free did not differ in a statistically significant way depending on seizure type (Fisher’s exact = 4.748, *p* = 0.316), sex (χ^2^ = 0.528, *p* = 0.468), age (Mann–Whitney U = 1227.5, *p* = 0.122), ASD number (Mann–Whitney U = 1258.0, *p* = 0.153) or seizure frequency (Mann–Whitney U = 1401.0, *p* = 0.608). Among written reasons for declining vaccination, individual respondents indicated that “the vaccine against COVID-19 is not yet fully explored for serious side-effects”, “there was little time for comprehensive research of the vaccine”. They also doubt that the vaccine is effective or know someone who had “severe complications” or “went to an intensive care unit” after vaccination against other infectious diseases. From the eight participants who already had COVID-19, two would agree to be vaccinated, three would refuse because of natural immunity and three—because of potential side-effects of the vaccine.

A binary logistic regression model (Table 3) identified that beliefs of vaccines being safe and being the only way to gain immunity without acquiring the infectious disease were associated with the willingness to be vaccinated (odds ratio (OR) = 7.90, 95% confidence interval (CI) = 2.43–25.74 and OR = 3.91, 95% CI = 1.02–15.05, respectively). Being vaccinated against influenza in 2020 was also positively related to the willingness to be vaccinated against COVID-19 (OR = 9.17, 95% CI = 1.15–73.47).

Most often, the primarily selected source of information regarding COVID-19 vaccines was reported to be specialists in neurology or epileptology (57, 51.4%) and general practitioners (36, 32.4%).

## 4. Discussion

### 4.1. People with Epilepsy and COVID-19

A similar number of both persons with epilepsy and caregivers responded to our survey. Persons with various seizure types and seizure frequencies were included, most used at least one ASD—it may therefore be assumed that the sample is representative of patients with active epilepsy. Our study could not confirm significant seizure exacerbation during the study period (as opposed to a period without national restrictions to mitigate the spread of COVID-19) among those who frequently experience seizures. This may be explained by the adaptation of the healthcare system to ensure the availability of ASDs as well as patient adaptation to lockdown measures during a second wave of the pandemic in December 2020. Further, the results indicate great compliance with the ASD regimen and reveal that patients almost never modified the use of their medication without supervision—this might have also prevented seizure exacerbation [2]. The ratio of confirmed cases of COVID-19 among the participants of our survey (eight cases in a sample of 111, or 7.2%) was similar to the one in the general population in Lithuania towards the end of the study (147,984 of confirmed cases in a population of 2,794,090 inhabitants, or 5.3%) [9]. However, COVID-19-related results (e.g., seizure exacerbation in one of the patients) are limited by a small sample size and the non-inclusion of severe or lethal COVID-19 cases, for which data are yet scant in the literature as well [6,10].

### 4.2. Vaccine Hesitancy among Persons with Epilepsy and Their Caregivers

Vaccination programs against COVID-19 started in various countries towards the end of 2020—with the emergence of effective COVID-19 vaccines, there still remain societal phenomena, namely vaccine hesitancy, that could significantly reduce the efficacy of COVID-19 mass-vaccination programs [11]. They may also remain a hurdle for the prevention of infectious diseases or mitigation of new pandemics in the future. Vaccine hesitancy is defined as a “delay in acceptance or refusal of vaccination despite availability of vaccination services” [12]. It is an ever-changing phenomenon that is influenced by social and cultural factors, individual or group opinions and vaccine-specific features. In our survey, we intentionally did not name a vaccine produced by a specific pharmaceutical company that would be used to mitigate the impact of COVID-19—it was presumed that any of the vaccines would be approved for use by the European Medicines Agency (EMA) and therefore meet the required safety and efficacy criteria. The EMA authorized one of the COVID-19 vaccines for use in the European Union during the study period and subsequent mass-vaccination was planned in the first half of 2021 (the U.S. Food and Drug Administration had already approved two COVID-19 vaccines as well) [13,14,15]. Despite widespread mediatization of the successful development of COVID-19 vaccines, most respondents in our study were unwilling to accept the vaccine against COVID-19, especially because of doubts of its safety and efficacy. The speed of vaccine development also became a source of uncertainty, as expressed by several of the participants. Rapid vaccine approvals have already been noted to beget skepticism in the general population and among healthcare workers [11]. Our findings indicate higher vaccine hesitancy than in studies from healthy populations (including results from polls in Lithuania that were commissioned by the Government, unpublished), where up to 60–70% of all respondents would likely agree to be vaccinated against COVID-19 [16,17]. Finally, around a third of adult patients unwilling to be vaccinated thought they could not get the vaccine because of epilepsy. This might reflect a perception that, in general, vaccines are associated with seizures (e.g., such as those containing measles) [18,19,20,21].

A binary logistic regression analysis revealed that a general belief in vaccine safety and an understanding of how vaccines function (i.e., that they provide immunity without causing an infectious disease) greatly increases the odds ratio that the study participant would prefer vaccination. Further, being vaccinated against influenza in 2020 was also associated with vaccine acceptance, as has already been noted in other settings [22,23]. In Lithuania, influenza vaccines are dispensed in outpatient clinics upon referral by general practitioners and are free of charge for persons at risk of complications (e.g., ≥65 years, pregnant women, patients with chronic conditions). However, no widespread informative campaigns are used to promote the vaccine.

We asked to provide a binary and definite (yes/no) answer about the will to be vaccinated—some persons, however, could still be undecided and swayed towards vaccination if additional information was provided [24]. While an anonymous survey form is advantageous for collecting honest opinions at a certain moment, it could not correlate with factual agreement to be vaccinated in real circumstances. Given that over half of all participants would first consult a neurologist to obtain explanations concerning the COVID-19 vaccine, it is essential for epilepsy specialists to be ready and willing to explain the mechanisms of vaccines and substantiate claims of vaccine safety and efficacy for their patients. A positive outlook towards vaccination among neurologists in Lithuania has been reported earlier [25]. Advances in telehealth, such as web-based informational material, could further enhance neurologist involvement in vaccination campaigns and counteract the influence of misleading information (e.g., in social networks) and increase vaccine uptake among PWE [26,27,28].

### 4.3. Study Limitations

Our study was cross-sectional and based online, therefore, limited because of non-respondent bias as well as inclusion of only those patients and caregivers who are members or followers of online epilepsy groups. Further, there were no mass-vaccination and related awareness campaigns in the country (before or during the study period), which could lead to an underestimation of the COVID-19 vaccine uptake potential among study participants. As vaccine hesitancy is context-specific and greatly depends on cultural, societal, and economic factors, the study needs to be replicated in different settings to substantiate our findings. Further studies could additionally investigate whether baseline knowledge about COVID-19 and societal factors (e.g., level of education) are associated with vaccine hesitancy among PWE. Finally, the lack of a control group does not allow for direct comparison with the general population and a relatively small sample size may reduce the generalizability of the results.

## 5. Conclusions

In the setting of an online survey, the majority of adult PWE and caregivers (representing mostly children with epilepsy) expressed reluctancy to be vaccinated against COVID-19. Prior vaccination history, positive outlook on the safety profile of vaccines and an understanding of how they function were the most important factors associated with the willingness to be vaccinated. Neurologists are seen as a primary source of information regarding COVID-19 vaccines and might have an important role in counteracting vaccine hesitancy among PWE.

## Figures and Tables

**Table 1:** Characteristics of the survey’s participants.

Survey Participants Who Provided Information	All (*n* = 111)	Patient Responses (*n* = 58)	Caregiver Responses (*n* = 53)	*p* Value
Percentage from all respondents	100	52.25	47.75	
Male/female (n, %)	44 (39.64)/67 (60.36)	13 (22.41)/45 (77.59)	31 (58.49)/22 (41.51)	<0.0001 **
	**n, % or median, range**	**n, % or median, range**	**n, % or median, range**	
Age (years)	25 (1–70)	35 (19–60)	8 (1–70) ^†^	<0.0001 **
Seizure type				0.005 *
Focal	49 (44.14)	33 (56.9)	16 (30.19)	
Generalized (“whole-body” seizures)	37 (33.33)	18 (31.03)	19 (35.85)	
Generalized (absence or myoclonic seizures)	10 (9.01)	3 (5.17)	7 (13.21)	
Other	10 (9.01)	1 (1.72)	9 (16.98)	
Unknown	5 (4.5)	3 (5.17)	2 (3.77)	
**Current seizure frequency (December 2020)**				0.261
Several times per day	14 (12.61)	2 (3.45)	12 (22.64)	
Several times per week	18 (16.22)	9 (15.52)	9 (16.98)	
Several times per month	25 (22.52)	20 (34.48)	5 (9.43)	
Several times per year	22 (19.82)	10 (17.24)	12 (22.64)	
Once per year	7 (6.31)	4 (6.90)	3 (5.66)	
Less than once every year	20 (18.02)	10 (17.24)	10 (18.87)	
No seizures for 5 years or longer	5 (4.50)	3 (5.17)	2 (3.77)	
**Seizure frequency during the summer of 2020**				0.400
Several times per day	17 (15.32)	5 (8.62)	12 (22.64)	
Several times per week	14 (12.61)	6 (10.34)	8 (15.09)	
Several times per month	27 (24.32)	21 (36.21)	6 (11.32)	
Once per month	6 (5.41)	2 (3.45)	4 (7.55)	
Once	13 (11.71)	6 (10.34)	7 (13.21)	
None	34 (30.63)	18 (31.03)	16 (30.19)	

n—number of patients, ^†^—the six adults represented by their caregivers were 18, 25, 27 (three patients), and 70 years old, *—*p* < 0.05, **—*p* < 0.0001.

**Table 2:** Study participants’ outlook on vaccination.

Survey Participants Who Provided Information	All (*n* = 111)	Patient Responses (*n* = 58)	Caregiver Responses (*n* = 53)	*p* Value
	**n, % or median, range**	**n, % or median, range**	**n, % or median, range**	
How much (from 1 to 10) are you waiting for a COVID-19 vaccine to arrive?	5 (1–10)	5 (1–10)	5 (1–10)	0.567
Vaccination against influenza before the COVID-19 pandemic	18 (16.22)	12 (20.69)	6 (11.32)	0.181
Vaccination against influenza in 2020	15 (13.51)	7 (12.07)	8 (15.09)	0.641
Vaccination against Pneumococcal infections in 2020	2 (1.8)	0 (0)	2 (3.77)	0.226
Acceptance of free COVID-19 vaccination	45 (40.54)	27 (46.55)	18 (33.96)	0.177
Acceptance of non-free COVID-19 vaccination	27 (24.32)	14 (24.14)	13 (24.53)	0.962
**Reasons for declining vaccination (n, % of those not accepting free vaccination)**				
I believe that vaccines cause COVID-19	6 (9.09)	4 (12.9)	2 (5.71)	0.408
I believe that vaccines may have long-term side effects	36 (54.55)	16 (51.61)	20 (57.14)	0.652
I do not believe that the vaccine is effective to stop the spread of COVID-19	27 (40.91)	11 (35.48)	16 (45.71)	0.399
I believe that I cannot be vaccinated because of epilepsy	19 (28.79)	11 (35.48)	8 (22.86)	0.258
Because of the price of the vaccine (if it was paid)	2 (3.03)	2 (6.45)	0 (0)	0.217
**Agreement with statements (n, % selected as true)**				
Vaccines are safe for most people and do not cause long-term side effects or complications	59 (53.15)	34 (58.62)	25 (47.17)	0.227
Vaccines may cause the infectious disease they aim to prevent	51 (45.95)	23 (39.66)	28 (52.83)	0.164
Vaccination is the only way to gain immunity apart from acquiring the disease itself	71 (63.96)	43 (74.14)	28 (52.83)	0.020 *
Vaccination is also useful for healthy people with no existing disease	76 (68.47)	39 (67.24)	37 (69.81)	0.771
There is a natural decrease of viral infections, regardless of the use of vaccines	44 (39.64)	23 (39.66)	21 (39.62)	0.997

*—*p* < 0.05.

**Table 3:** A binary logistic model (*n* = 111, Nagelkerke R^2^ = 0.556, *p* < 0.0001) with willingness to be vaccinated for free being the dependent variable (“yes” = 1, “no” = 0).

Independent Variable (“Yes” = 1, “No” = 0)	Coefficient	*p* Value	Odds Ratio (95% Confidence Interval)
Vaccines are safe for most people and do not cause long-term side effects or complications	2.067	0.001 *	7.90 (2.43–25.74)
Vaccines may cause the infectious disease they target	−1.942	0.002 *	0.14 (0.04–0.49)
Vaccination is the only way to gain immunity apart from acquiring the disease itself	1.365	0.047 *	3.91 (1.02–15.05)
Vaccination is also useful for healthy people with no existing disease	1.072	0.153	2.92 (0.67–12.73)
There is a natural decrease of viral infections, regardless of the use of vaccines	0.538	0.399	1.71 (0.49–5.99)
Used to be vaccinated against influenza before the COVID-19 pandemic	−0.159	0.854	0.85 (0.16–4.65)
Vaccination against influenza in 2020	2.216	0.037 *	9.17 (1.15–73.47)
COVID-19 infection	−0.880	0.383	0.42 (0.06–2.99)
Constant	−3.032	0.001 *	0.05 (n/a)

*—*p* < 0.05, n/a—not applicable.

## Data Availability

Raw study data are available from the authors upon reasonable request.

## Supplementary Materials

The following are available online at https://www.mdpi.com/article/10.3390/ijerph18084374/s1, File S1: Translated questionnaire used in the study.
