# Supplementary material for: Factors Associated with COVID-19 Vaccine Hesitancy among People with Epilepsy in Lithuania
Source: Int J Environ Res Public Health. 2021 Apr 20;18(8):4374. doi: 10.3390/ijerph18084374 (PMC8074300; doi:10.3390/ijerph18084374)
Supplement: Supplementary file 1 [file ijerph-18-04374-s001.zip › ijerph-1191600-supplementary.pdf]

Translated questionnaire from Lithuanian

All items were spread throughout the page for comfortable reading in the platform "Google Forms" (Google, Inc.)

Your status ☐Person with epilepsy ☐Caregiver (please provide information about the person you care for)

General questions

Age___ **Sex** ☐Male ☐Female **Seizure type** ☐Focal (episodes without falling and having whole-body seizures that may include other symptoms) ☐ Generalized ("whole-body" seizures, you usually do not feel the seizure starting) ☐Generalized (absence or myoclonic seizures) ☐Unknown -Other___ **Number of antiseizure drugs (ASDs) used** ☐0 ☐1 ☐2 ☐3 ☐4 or more ASDs –Fill-in ASDs used____

Have you changed your ASDs or their dosage in 2020 as recommended by your physician? ☐Yes, the dosage was increased or new ASDs were added ☐Yes, one ASD was changed into another ☐Yes, the dosage was decreased or some ASD was discontinued ☐No, I used ASDs as usual ☐I do not use ASDs

Have you changed your ASDs or their dosage in 2020 yourself? ☐Yes, I increased the dosage or added new ASDs ☐Yes, I changed one ASD into another ☐Yes, I decreased the dosage or discontinued some ASDs ☐No, I used ASDs as usual ☐I do not use ASDs

How frequently do you currently experience seizures? ☐Several times per day ☐Several times per week ☐Several times per month ☐Several times per year ☐Once per year ☐Less than once every year ☐I do not have seizures for 5 years or longer

How frequently did you experience seizures in summer of 2020? ☐Several times per day ☐Several times per week ☐Several times per month ☐Once per month ☐Once ☐None

COVID-19

Were you confirmed to have COVID-19? ☐Yes ☐No -If yes, which month? ___(selection from a list)

Which of these symptoms of COVID-19 did you experience? ☐Fever ☐Dry cough ☐Tiredness ☐Aches and pains ☐Sore throat ☐Diarrhea ☐Conjunctivitis (red eye, lacrimation, itching) ☐Headache ☐Loss of taste or smell ☐A rash on skin, or discoloration of fingers or toes ☐Difficulty breathing or shortness of breath ☐Chest pain or pressure ☐Inability to speak or move ☐Stroke ☐Loss of consciousness (but not because of a seizure) -Other___

Did the frequency of your seizures change when having COVID-19? ☐Yes, increase by two times or more ☐Yes, increase by up to two times ☐No ☐Yes, decrease by up to two times ☐Yes, decrease by two times or more

Presumed place of infection ☐A public place (shop, mass event etc.) ☐Home (infection from family members) ☐Home (infection from a non-family member) ☐Workplace or education institution ☐Healthcare institution (hospital, rehabilitation center) ☐Healthcare institution (outpatient clinic) ☐Unknown -Other___

Who provided information concerning COVID-19 after confirming the infection? ☐A general practitioner ☐A specialist from the National Center for Public Health ☐A specialist in neurology (general) ☐A specialist in epileptology ☐A physician in an ambulance ☐A physician at the emergency department ☐No one -Other___

Were you hospitalized because of COVID-19? ☐Yes ☐No **If yes, for how many days?**___

Were you in an intensive care unit because of COVID-19? ☐Yes ☐No ☐I don’t know

Did you change your ASD use (drugs, dose or rate) when having COVID-19? ☐Yes ☐No

Do you have any comorbidities? ☐Disorders of heart and circulation ☐Lung diseases ☐Diabetes mellitus ☐Chronic kidney diseases ☐Chronic liver/pancreatic conditions -Other___

Vaccines

How much (from 1 to 10) are you waiting for a COVID-19 vaccine to arrive to Lithuania?

	1	2	3	4	5	6	7	8	9	10	
Not at all	☐	☐	☐	☐	☐	☐	☐	☐	☐	☐	Very much

Were you used to vaccinating against influenza before the pandemic? ☐Yes ☐No

Were you vaccinated against influenza in 2020? ☐Yes ☐No

Were you vaccinated against Pneumococcal infections in 2020? ☐Yes ☐No

Would you accept to be vaccinated against COVID-19 for free? ☐Yes ☐No

Would you accept to be vaccinated against COVID-19 if the vaccine was not free? ☐Yes ☐No

Why would you not accept to be vaccinated against COVID-19? ☐I believe that vaccines cause COVID-19 ☐I believe that vaccines may have long-term side effects ☐I do not believe that the vaccine is effective to stop the spread of COVID-19 ☐I believe that I cannot vaccinate because of epilepsy ☐Because of the price of the vaccine (if it was paid) -Other___

Please indicate, whether you believe these statements to be true or false:

Vaccines are safe for most people and do not cause long-term side effects or complications ☐True ☐False

Vaccines may cause the infectious disease they aim to prevent ☐True ☐False

Vaccination is the only way to gain immunity apart from acquiring the disease itself ☐True ☐False

Vaccination is also useful for healthy people with no existing disease ☐True ☐False

There is a natural decrease of viral infections, regardless of the use of vaccines ☐True ☐False

What would be your primary source of information considering COVID-19 vaccines? ☐A general practitioner ☐A pharmacist ☐A specialist in neurology (general) ☐A specialist in epileptology ☐Friends and family members ☐Internet ☐Television -Other____
